# A 28,000 Years Old Cro-Magnon mtDNA Sequence Differs from All Potentially Contaminating Modern Sequences

**DOI:** 10.1371/journal.pone.0002700

**Published:** 2008-07-16

**Authors:** David Caramelli, Lucio Milani, Stefania Vai, Alessandra Modi, Elena Pecchioli, Matteo Girardi, Elena Pilli, Martina Lari, Barbara Lippi, Annamaria Ronchitelli, Francesco Mallegni, Antonella Casoli, Giorgio Bertorelle, Guido Barbujani

**Affiliations:** 1 Dipartimento di Biologia Evoluzionistica, Università di Firenze, Firenze, Italy; 2 Dipartimento di Biologia ed Evoluzione , Università di Ferrara, Ferrara, Italy; 3 Centro di Ecologia Alpina Fondazione Edmund Mach, Viote del Monte Bondone, Trento, Italy; 4 Dipartimento di Biologia, Università di Pisa, Pisa, Italy; 5 Dipartimento di Dipartimento di Scienze Ambientali , Università di Siena, Siena, Italy; 6 Dipartimento di Chimica Generale e Inorganica, Chimica Analitica, Chimica Fisica, Università di Parma, Parma, Italy; University of Utah, United States of America

## Abstract

**Background:**

DNA sequences from ancient speciments may in fact result from undetected contamination of the ancient specimens by modern DNA, and the problem is particularly challenging in studies of human fossils. Doubts on the authenticity of the available sequences have so far hampered genetic comparisons between anatomically archaic (Neandertal) and early modern (Cro-Magnoid) Europeans.

**Methodology/Principal Findings:**

We typed the mitochondrial DNA (mtDNA) hypervariable region I in a 28,000 years old Cro-Magnoid individual from the Paglicci cave, in Italy (Paglicci 23) and in all the people who had contact with the sample since its discovery in 2003. The Paglicci 23 sequence, determined through the analysis of 152 clones, is the Cambridge reference sequence, and cannot possibly reflect contamination because it differs from all potentially contaminating modern sequences.

**Conclusions/Significance:**

The Paglicci 23 individual carried a mtDNA sequence that is still common in Europe, and which radically differs from those of the almost contemporary Neandertals, demonstrating a genealogical continuity across 28,000 years, from Cro-Magnoid to modern Europeans. Because all potential sources of modern DNA contamination are known, the Paglicci 23 sample will offer a unique opportunity to get insight for the first time into the nuclear genes of early modern Europeans.

## Introduction

The anatomically-archaic Europeans, the Neandertal people, are documented in the fossil record from approximately 300,000 to 30,000 years ago. Around 45,000 years ago, anatomically-modern humans of the Cro-Magnoid type expanded in Europe from the Southeast. Neandertals coexisted with them for between 1,000 to 10,000 thousand years, depending on the region [1], but eventually their skeletons disappeared from the fossil record. Individuals of intermediate morphology have not been observed. With the possible exception of one 25,000 years old child [2], all known specimens in the relevant time interval can be classified without ambiguity either as Neandertals or Cro-Magnoids.

The interpretation of these findings is not straightforward. Under the so-called Out-of-Africa model, Neandertals are considered to be extinct, and modern Europeans are regarded as descending exclusively from Cro-Magnoids who replaced Neandertals in the course of their expansion from Africa [3]. Conversely, recent versions of the alternative, multiregional model, propose that Neandertals gave a limited, but non-negligible, contribution to the gene pool of modern Europeans by admixing with Cro-Magnoids (e.g. [4]–[6]). Analyses of morphological traits [7], ancient Neandertal DNA [8], [9], and modern DNA diversity [10]–[12] are generally regarded as supporting a recent African origin of modern humans [13], without substantial Neandertal contribution, if any at all. In particular, mtDNA sequences from all studied Neandertals fall out of the range of modern variation and show no particular relationship with modern European sequences [9], [14]. However, it is clearly impossible to rule out any degree of reproductive interaction between the two groups. As a consequence, the possibility has been raised that admixture did occur, but the early Europeans of modern anatomy were not too different genetically from Neandertals, or else that most Neandertal haplotypes were lost through a process of lineage sorting, i.e. by genetic drift [5].

To clarify the evolutionary relationships between the two anatomically-distinct groups that coexisted in Upper Paleolithic Europe, data on DNA variation in Cro-Magnoids are of course extremely important. At present, only two Cro-Magnoid sequences, both from Paglicci in Southern Italy, have been published. Both of them fall within the range of modern mtDNA variation, thus differing sharply from all known Neandertal sequences, and both belong to fossil specimens from which Neandertal-specific primers failed to amplify mtDNA [15]. Serre et al. [16] confirmed that Cro-Magnoid mtDNAs could not be amplified using Neandertal-specific primers, but argued that the Paglicci sequences, as well as all ancient sequence that appear modern, cannot be considered reliable because contamination of ancient samples by modern DNA can be proved, but absence of contamination cannot.

Undetected contamination is doubtless a serious problem in ancient human DNA study, as shown by the presence of modern human DNA in samples that should not naturally contain it [16]–[20]. However, the fact that such contamination can and does occur does not imply that it cannot be recognized [21]. Presumably, modern DNA tends to permeate in the pulp cavity of the teeth through dentinal tubules, and in the bone through the Haversian system [22], although possibly not reaching the osteocytes [18], [19]. The main causes of contamination are the direct handling and washing of the specimens, most likely in the phase immediately after excavation [22], [23].

In this study we had the unique opportunity to characterize genetically a Cro-Magnoid individual, Paglicci 23, whose tafonomic history is perfectly known. As a consequence, we could monitor all possible contaminations from the individuals who manipulated the sample. In this way, testing for contamination meant comparing the sequence obtained from the Paglicci 23 bones with the sequences of all modern people who touched them, and not with generic and hard-to define modern sequences. We showed that: (i) the mitochondrial sequence inferred from the analysis of the Paglicci 23 mtDNA hypervariable region I (HVR I) cannot possibly be due to contamination by anybody who manipulated the sample ever since its discovery in 2003, and (2) this 28,000 years old sequence is still common in Europe, and is the Cambridge reference sequence (CRS).

## Results and Discussion

**Figure 1:**
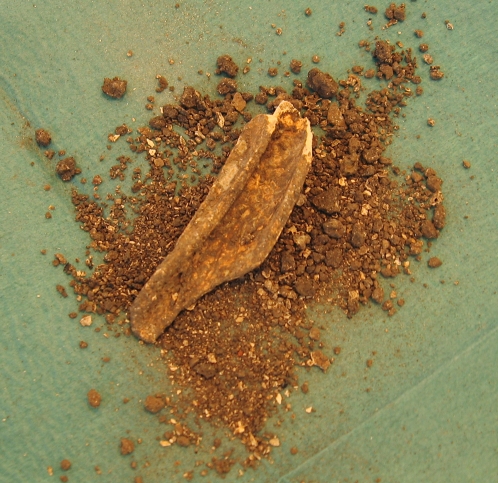
Tibia fragment of the Paglicci 23 specimen. DNA was extracted from this fragment and from skull splinters, and all extracts yielded the same HVR I sequence.

The fragmentised remains (tibia, skulls, jaw and maxilla) of a Cro-Magnon individual, named Paglicci 23, were excavated by F.M in 2003 from the Paglicci cave, Southern Italy. Radiocarbon tests dated the layer to 28.100 (+/−350) years ago [24]. Because of its fragmentary nature, the sample was neither restored nor studied from the morpho-anatomical point of view. Therefore, no contamination could possibly be introduced at these stages by direct handling and washing. The remains were deposited in the storage room at controlled temperature in the Department of Archaeology, University of Pisa. In 2005 three splinters, a piece of tibia (Figure 1) and two pieces of skull, were moved to the ancient DNA laboratory at the University of Florence. In the course of the whole process, from excavation of the remains to genetic typing, only seven persons had any contacts with the sample, namely six of us (F.M, S.V, A.M., E.Pi., M.L., and D.C.) and Carles Lalueza-Fox (hereafter: C.L.) who replicated the sequence at the University Pompeu Fabra, Barcelona.

The degree of racemization of three amino acids, aspartic acid, alanine, and leucine, provides indirect evidence as for the presence in an ancient sample of amplifiable DNA. In particular, DNA is expected to be too degraded for amplification when the D/L for Asp is greater than 0.08 [25]. As a preliminary test of macromolecule preservation, we measured the stereoisomeric D/L ratio for these amino acids. The observed values, all of them compatible with good preservation of biological macromolecules in the sample, were D/L Asp 0.0479, D/L Glu 0.0104 D/L, Ala 0.0092. The global amino acid content was 42,589 parts per million, and endogenous DNA was successfully ampified from Pleistocene remains when this value was higher than 30,000 parts per million [16].

Quantitative PCR showed a relatively large amount of mtDNA molecules in the Paglicci 23 fossil, approximately 2300. Contamination, usually detected when different sequences are observed in different cloned products, is considered unlikely if the number of PCR template molecules is >1,000 [26]. We thus proceeded in the analysis by initially sequencing a total of 144 clones (Table S1), respectively 64, 32 and 48 for the three regions in which the HVR I was divided. Reproducible mtDNA sequences corresponding to positions 16024–16383 of the published reference sequence CRS [27] were obtained in the Florence laboratory from the tibia and from a skull fragment of Paglicci 23. No contamination was observed in the extractions and PCR blanks. Amplification of long DNA fragments, unusual for ancient DNA, was not observed. The analysis was repeated in Barcelona, using a tibia fragment; the consensus sequence obtained from 8 clones covering the region between nt 16245 to nt 16349 was identical to that obtained in Florence. On the contrary, no PCR product was observed when we attempted to amplify the DNA extracts using two pairs of Neandertal-specific primers.

As is common in studies of ancient DNA, when comparing sequences across clones we observed single nucleotide substitutions occurring in one or a few clones (Table S1), on average 3.9 every 1,000 bp. In addition, a C to T change was observed in 27 out of 56 clones at nt 16274. In principle, differences of this kind across clones may be due to three factors, namely: (1) sequence heterogeneity due to the presence of exogenous, contaminating DNA, (2) post-mortem DNA damage, and (3) *Taq*-polymerase errors or cloning artefacts. We tested separately for the possible effects of the first two factors upon our specimen.

To track down any possible modern contaminations, the mtDNAs of the seven authors who to any extent manipulated the sample were genotyped. All these sequences (Table 1) differ from the Paglicci 23′s consensus mtDNA sequence. However, two of (F.M and C.L) have a T at nt 16274. Therefore, variation across clones at that site might have meant that either investigator left his DNA on the sample, although C.L. had no contacts with the material at the stage at which clones F2.1 through F2.13 and F3.1 through F3.15 were genotyped.

**Table 1:** Mitochondrial HVR1 variation in the seven researchers that have been in physical contact with the samples.

Researcher	Task	HVR1 haplotype
F.M	Excavation	16069 T, 16126 C, 16278 T, 16294 T, 16366 T
S.V	Laboratory analysis	16311 C
A.M	Laboratory analysis	16274 A 16311 C
M.L	Laboratory analysis	16261 T, 16311 C
E.Pi	Laboratory analysis	16096 A, 16126 C, 16145 A, 16189 C, 16231C, 16260 T, 16261 T,
C.L.	Laboratory analysis	16126 C, 16294 T, 16296 T, 16304 C
D.C.	Laboratory analysis	16193 T, 16278 T

Post-mortem DNA damage generally occurs in the form of double-strand breaks, or other modifications severe enough to prevent enzymatic replication of the DNA molecule. Had this happen, we would have been unable to amplify the DNA. However, hydrolytic deamination and depurination may also occur, resulting in apparent changes of the nucleotide sequence. Although post-mortem damages of this kind are unlikely to severely bias the results when the initial template molecules exceed 1000 [26] as is the case for Paglicci 23, to correct for such possible post-mortem damages, a third DNA extract was treated with Uracyl-N-Glycosidase (UNG) [17], and independently resequenced. The 35 sequences thus obtained (clones F 4.1 through F4.20, and F5.1 through F 5.15) contain no nucleotide substitutions with respect to the CRS, including nt 16274 (Table S1). As a consequence, we concluded that the sequence obtained from the Paglicci 23 specimen is the CRS, and that heterogeneity across clones at nt 16274 reflects DNA damage due to deamination of the original cytosine and successive amplification of the damaged DNA fragment(s). The rate of nucleotide misincorporation suggests that the DNA templates were indeed damaged (3.9 substitutions every 1,000 bp within the HVRI), but after UNG treatment at least 82% of the clones showed the same consensus nucleotide at each position (Table S1).

**Figure 2:**
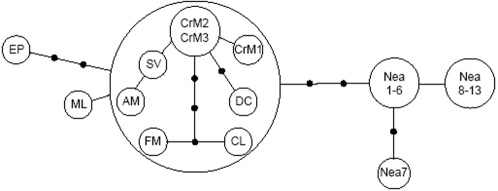
Genetic relationships among the Paglicci 23 and other relevant mtDNA sequences. The network summarizes mtDNA HVR I variation in 13 Neandertals (Nea1 to Nea13) , three Cro-Magnons (CrM1 to CrM3), and seven modern humans who manipulated the Cro-Magnons specimens (six authors of this paper and Carles Laueza-Fox, designated by their initials).

The relationship between the Paglicci 23 sequence, the available Cro-Magnon and Neandertal sequences, and all the sequences from the seven individuals who manipulated the Cro-Magnons specimen, are summarized in Figure 2. The backbone of the network is based on the 31bp region for which we had complete overlap among all sequences, and was estimated by a statistical parsimony method [28], as implemented in the software TCS [29]. A sub-network was also reconstructed for a set of eight individuals relevant to this study using the entire fragment of 360 bp.

Previous genetic data on Cro-Magnoids [15], although generated under the most stringent available criteria, were considered problematic by some authors [16], [30], because the mtDNA sequences obtained correspond to sequences also observed in modern individuals. For most ancient human samples, rigorous application of this criterion would render the study of Cro-Magnoid DNA practically impossible, because it is impossible to rule out any contamination from generic unknown individuals. However, it is possible to test for the occurrence in the extract of known potential contaminating sequences; for the Paglicci 23 fossil we had this opportunity, and we found that none of these modern sequences is equal to the sequence obtained from the fossil extracts. Since we used different sets of overlapping primers pairs to amplify the fragment included between nucleotide 16024 and 16383, it seems highly unlikely that the sequence obtained was a chimera artefact.

Therefore, at this stage it is safe to conclude that at least one Cro-Magnoid mtDNA sequence, for which contamination can be ruled out with a high degree of confidence, falls well within the range of modern human variation. This does not prove, but at least indirectly suggests, that the previously published Cro-Magnoid sequences [15], both documented in the modern human gene pool, may be genuine [31]. At any rate, the finding of the Cambridge Reference Sequence in Paglicci 23 shows that one of today's mtDNA variants has been present in Europe for at least 28,000 years, and that modern and archaic anatomical features appear associated with mtDNA sequences that can be classified, respectively, as modern and non-modern. Because no HVR I sequence similar to the Neandertals' has been described in more than 4800 Europeans studied so far [32], models whereby Neandertals were part of the genealogy of current Europeans are at odds with the data, at least as far as maternal inheritance is concerned. In our opinion, the burden of the proof is now on those who maintain that Neandertals might have contributed to the modern gene pool.

So far, the study of ancient nuclear DNA in humans has been severely limited by the difficulty to ascertain whether the DNA sequences obtained are really endogenous to the specimen. This study shows that it is possible to test for DNA authenticity, provided the people who manipulate the sample from the moment of excavation are carefully recorded and their DNAs typed. Therefore, Paglicci 23 (as well as other remains studied under comparable conditions in the future) promises to be a valuable source of information on DNA diversity in the past, and can pave the ground for a more exhaustive understanding of human evolutionary history.

## Materials and Methods

### DNA extraction

All DNA-preparation and extraction methods followed strictly specific ancient DNA requirements [33]. DNA was extracted in two laboratories, in Florence and Barcelona, in facilities exclusively dedicated to ancient DNA work. All DNA extractions and PCR set up were carried out in physically separated spaces from those in which PCR cyclings and post-PCR analysis was conducted. Full-body suits, disposable masks and gloves were worn throughout and were changed frequently, and pipettors were UV-irradiated in between use. All DNA extractions and PCR reactions included negative controls, and all steps of the analysis were replicated at least twice in each laboratory. To test for preservation of other macromolecules as an indirect evidence for DNA survival [26] we estimated the degree of aminoacid racemization, in each sample, using approximately 5 mg of tibia and skull, powdered following the procedures described in [25]. We quantified the amount of target DNA by Real Time (RT) PCR. PCR products were cloned, 152 clones were sequenced, and the sequences thus obtained were aligned and compared across clones. After extraction, UNG treatment were performed on a third skull fragment in order to verify whether C to T changes (nt 16294 ) observed in some clones represented postmortem damage or contamination [17].

To prevent contamination from prior handling, the outer layer of bones was removed with a rotary tool, and the fragments were briefly soaked in 10% bleach. Both samples were then irradiated (1 hour under UV light) and powdered. DNA was extracted by means of a silica-based protocol [15]. At least two independent extracts were obtained from each remain. Multiple negative controls were included in each extraction.

### UNG treatment

Uracil bases caused by the hydrolytic deamination of cytosines were excised by treating 10 μl of DNA extracted from both samples with 1U of Uracil-N-Glycosylase (UNG) for 30 min at 37°C. UNG reduces sequence artefacts caused by this common form of post-mortem damage, resulting in apparent C to T/G to A mutations and subsequent errors in the sequence results [17]. After this treatment, the extract was subjected to the same PCR Cloning and sequencing conditions as described above.

### Quantification of DNA Molecules

Real-time PCR amplification was performed using Brilliant® SYBR® Green QPCR Master Mix (Stratagene) in MX3000P (Stratagene), using 0.5μM of appropriate primers (forward primer located at H 16107 and reverse primer located at L 16261. Thermal cycling conditions were 95°C for 10 min, 40 cycles at 95°C for 30 s, 53°C for 1 min and 72°C for 30 s, followed by SYBR® Green dissociation curve steep. Ten-fold serial dilutions of the purified and quantified standard were included in the experiment to create the standard curve in order to know the number of initial DNA molecules in the samples

### Amplification of mt DNA

Two μl of DNA extracted from the bone were amplified with this profile: 94°C for 10 min (Taq polymerase activation), followed by 50 cycles of PCR (denaturation , 94°C for 45 sec, annealing, 53°C for 1 min and extension, 72°C for 1 min) and final step at 72°C for 10 min. The 50 μl reaction mix contained 2 U of AmpliTaq Gold (Applied Biosystems), 200 μM of each dNTP and 1 μM of each primer. The 360 bp long HVR-I was subdivided in three overlapping fragments using the following primer pairs: L15995/H16132; L16107/H16261; L16247/H16402. Each extract was amplified at least twice. Since overlapping primers were used throughout the PCR amplifications, it is highly unlikely that we amplified a nuclear insertion rather than the organellar mtDNA. Reactions conditions in replay analysis were described in [34], except for the sequences primers that we report as follows: 5′ACTATCACACATCAACTGC 3′; 5′ATGGGGACGAGAAGGGATTT 3′.

### Cloning and Sequencing

PCR products were cloned using TOPO TA Cloning Kit (Invitrogen) according to the manufacturer's instructions. Screening of white recombinant colonies was accomplished by PCR, transferring the colonies into a 30 μl reaction mix (67 mM Tris HCl [pH 8.8], 2 mM MgCl_2_, 1 μM of each primer, 0.125 mM of each dNTP, 0,75 units of Taq Polymerase) containing M13 forward and reverse universal primers. After 5 min at 92° C, 30 cycles of PCR (30 sec at 90°C, 1 min at 50°C, 1 min at 72°C) were carried out and clones with insert of the expected size were identified by agarose gel electrophoresis. After purification of these PCR products with Microcon PCR devices (Amicon), a volume of 1,5 μl was cycle-sequenced following the BigDye Terminator kit (Applied Biosystems) supplier's instructions. The sequence was determined using an Applied BioSystems 3100 DNA sequencer.

### “Long” amplificate detection

Appropriate molecular behaviour was also tested by amplification of longer mtDNA fragments (443 bp and 724 bp), which have been reported as very unusual for ancient DNA. PCR conditions were those described for mtDNA analysis above, primers used for 443 bp fragment were L15995 and H16401, while for 724 bp fragment primers used were L16247 and H00360.

### Amplification with Neandertal-specific primers

Amplifications of the Paglicci extracts with two pairs of Neandertal-specific primers (L16,022-NH16,139 and NL16,263/264-NH16,400, [14]) were also attempted. 50 μl of DNA were amplified with the following profile: 94°C for 10 min and 45 cycles of a denaturation (94°C for 45 sec), annealing (57°C for 1 min for the first couple and 59°C for 1 min for the second couple) and extension step (72°C for 1 min). The 50 μl reaction mix contained 2 U of AmpliTaq Gold polymerase and 1× reaction buffer (Applied Biosystems), 200 μM of each dNTP, 1.5mM MgCl2, 1 μM of each primer.

### Extractions amplifications and sequencing of modern DNA

MtDNA genotypes of all individuals who had any contacts with the specimen were either known in advance (M.L., D.C. and C.L.F: [21]), or determined in the Laboratory at Viote Trento (S.V., E.Pi., A.M., F.M.). Buccal cells were collected by oral brushes (Sterile Omni Swab or Sterile Foam Tipped Swabs, Whatman International Ltd., Maidstone, UK) and DNA was extracted using QIAmp1 DNA Mini Kit (QIAGEN, Hagen, Germany) according to manufacturer's instructions. The hypervariable region I (HVR1) of the mtDNA was determined by PCR amplification using the primers L15996 (5′CTCCACCATTAGCACCCAAAGC ′3) and H408 (5′ CTGTTAAAAGTGCATACCGCC ′3) (Table S1).

## Supporting Information

Table S1 Sequences of the clones obtained by amplifying the HVR I of mtDNA from the Paglicci 23 fossil. A dot indicates identity with respect to the Cambridge Reference Sequence, as modified by Ruiz-Pesini et al. [26], a letter indicates a nucleotide substitution. In the first column, labels designate clones sequenced, respectively, in the Florence (those beginning with an F) or Barcelona (those beginning with a B) laboratories. Bold type: clone sequences after UNG treatment. The sequences of the primers are also reported.
(0.09 MB DOC)
