# Supplementary material for: A 28,000 Years Old Cro-Magnon mtDNA Sequence Differs from All Potentially Contaminating Modern Sequences
Source: PLoS One. 2008 Jul 16;3(7):e2700. doi: 10.1371/journal.pone.0002700 (PMC2444030; doi:10.1371/journal.pone.0002700)
Supplement: Table S1 — Sequences of the clones obtained by amplifying the HVR I of mtDNA from the Paglicci 23 fossil. A dot indicates identity with respect to the Cambridge Reference Sequence, as modified by Ruiz-Pesini et al. [26], a letter indicates a nucleotide substitution. In the first column, labels designate clones sequenced, respectively, in the Florence (those beginning with an F) or Barcelona (those beginning with a B) laboratories. Bold type: clone sequences after UNG treatment. The sequences of the primers are also reported. (0.09 MB DOC) [file pone.0002700.s001.doc]

111111111111111111111111111111111111111111111111111111111111111111111111111111111111111111111111111111111111111111111111111111111111111111111111111111111111111111111111111111111111111111111111111111111111111111111111111111111111111111111111111111111111111111111111111111111111111111111111111111111111111111111111111111111111111111111111111111111111111111111111

666666666666666666666666666666666666666666666666666666666666666666666666666666666666666666666666666666666666666666666666666666666666666666666666666666666666666666666666666666666666666666666666666666666666666666666666666666666666666666666666666666666666666666666666666666666666666666666666666666666666666666666666666666666666666666666666666666666666666666666666

000000000000000000000000000000000000000000000000000000000000000000000000000011111111111111111111111111111111111111111111111111111111111111111111111111111111111111111111111111112222222222222222222222222222222222222222222222222222222222222222222222222222222222222222222222222222333333333333333333333333333333333333333333333333333333333333333333333333333333333333

222222333333333344444444445555555555666666666677777777778888888888999999999900000000001111111111222222222233333333334444444444555555555566666666667777777777888888888899999999990000000000111111111122222222223333333333444444444455555555556666666666777777777788888888889999999999000000000011111111112222222222333333333344444444445555555555666666666677777777778888

456789012345678901234567890123456789012345678901234567890123456789012345678901234567890123456789012345678901234567890123456789012345678901234567890123456789012345678901234567890123456789012345678901234567890123456789012345678901234567890123456789012345678901234567890123456789012345678901234567890123456789012345678901234567890123456789012345678901234567890123

TTCTTTCATGGGGAAGCAGATTTGGGTACCACCCAAGTATTGACTCACCCATCAACAACCGCTATGTATTTCGTACATTACTGCCAGCCACCATGAATATTGTACGGTACCATAAATACTTGACCACCTGTAGTACATAAAAACCCAATCCACATCAAAACCCCCTCCCCATGCTTACAAGCAAGTACAGCAATCAACCCTCAACTATCACACATCAACTGCAACTCCAAAGCCACCCCTCACCCACTAGGATACCAACAAACCTACCCACCCTTAACAGTACATAGTACATAAAGCCATTTACCGTACATAGCACATTACAGTCAAATCCCTTCTCGTCCCCATGGATGACCCCCCTCA

F.1.1 (MINUS 19BP)....................................................................................................C...............ATGAACTGGTGGACATCATGTAT

F.1.2 ....................................................................................................................

F.1.3 ....................................................................................................................

F.1.4 ....................................................................................................................

F.1.5 ....................................................................................................................

F.1.6 ....................................................................................................................

F.1.7 ....................................................................................................................

F.1.8 ....................................................................................................................

F.2.1 ....................................................................................................................

F.2.2 ....................................................................................................................

F.2.3 ....................................................................................................................

F.2.4 ...................................G................................................................................

F.2.5 ....................................................................................................................

F.2.6 .....................................................................CC.............................................

F.2.7 ....................................................................................................................

F.2.8 ....................................................................................................................

F.3.1 ....................................................................................................................

F.3.2 ....................................................................................................................

F.3.3 ....................................................................................................................

F.3.4 ....................................................................................................................

F.3.5 ....................................................................................................................

F.3.6 ....................................................................................................................

F.3.7 ....................................................................................................................

F.3.8 ....................................................................................................C...............

F.4.1 ....................................................................................................................

F.4.2 ...................................G................................................................................

F.4.3 ....................................................................................................................

F.4.4 ....................................................................................................................

F.4.5 ....................................................................................................................

F.4.6 ....................................................................................................................

F.4.7 ....................................................................................................................

F.4.8 ....................................................................................................................

F.1.1 ....................................................................................................................

F.1.2 ....................................................................................................................

F.1.3 ......................................................................C.............................................

F.1.4 ....................................................................................................................

F.1.5 ....................................................................................................................

F.1.6 ....................................................................................................................

F.1.7 ....................................................................................................................

F.1.8 ....................................................................................................................

F.2.1 ....................................................................................................................

F.2.2 ....................................................................................................................

F.2.3 ....................................................................................................................

F.2.4 ....................................................................................................................

F.2.5 ....................................................................................................................

F.2.6 ....................................................................................................................

F.2.7 ....................................................................................................................

F.2.8 ....................................................................................................................

F.3.1 ......................................................................C.............................................

F.3.2 ....................................................................................................................

F.3.3 ....................................................................................................................

F.3.4 ....................................................................................................................

F.3.5 ....................................................................................................................

F.3.6 ....................................................................................................................

F.3.7 ....................................................................................................................

F.3.8 ......................................................................C.............................C...............

F.4.1 ....................................................................................................................

F.4.2 ....................................................................................................................

F.4.3 ....................................................................................................................

F.4.4 ....................................................................................................................

F.4.5 ....................................................................................................................

F.4.6 ....................................................................................................................

F.4.7 ....................................................................................................................

F.4.8 ....................................................................................................................

F.1.1 GTATTTCGTACATTACTGCC....................................................................................................................GTTGATAGTGTGTAGTTG

F.1.2 ....................................................................................................................

F.1.3 ....................................................................................................................

F.1.4 ..-....................................-.....................................................T......................

F.1.5 .........-...........................G.-.....................................................T......................

F.1.6 .............................................................................................T......................

F.1.7 ....................................................................................................................

F.1.8 .....................................G.......................................................T......................

F.2.1 ....................................................................................................................

F.2.2 ....................................................................................................................

F.2.3 ....................................................................................................................

F.2.4 ..-.................................................................................................................

F.2.5 .........-...........................G..............................................................................

F.2.6 .............................................................................................T......................

F.2.7 ....................................................................................................................

F.2.8 .....................................G..............................................................................

F.3.1 ....................................................................................................................

F.3.2 ....................................................................................................................

F.3.3 ....................................................................................................................

F.3.4 ..-.................................................................................................................

F.3.5 .........-..........................................................................................................

F.3.6 .....................................G..............................................................................

F.3.7 ....................................................................................................................

F.3.8 ....................................................................................................................

F.4.1 ....................................................................................................................

F.4.2 ....................................................................................................................

F.4.3 ....................................................................................................................

F.4.4 ..-.................................................................................................................

F.4.5 .........-..........................................................................................................

F.4.6 ....................................................................................................................

F.4.7 ....................................................................................................................

F.4.8 ....................................................................................................................

ACTATCACACATCAACTGC............................A...................T......................................................AAATCCCTTCTCGTCCCCAT

B.1.2 ................................................T.T.......C............................................

B.1.3 .......................................................................................................

B.1.4 .......................................................................................................

B.1.5 .......................................................................................................

B.1.6 .......................................................................................................

B.1.7 .......................................................................................................

B.1.8 ................................................T.T.......C............................................

F.1.1 CAACTATCACACATCAACTGCAA-------------------------------.........................................................................................................(MINUS 20BP)

F.1.2 ----------------------------------------................................................................................................

F.1.3 -------------------------------.........................................................................................................

F.1.4 -------------------------------.........................................................................................................

F.1.5 ---------------------------------.......................................................................................................

F.1.6 -------------------------------.........................................................................................................

F.1.7 ..............................................T.........................................................................................

F.1.8 ..............................................T.........................................................................................

F.1.9 ----------------------------------------................................................................................................

F.1.10 ---------------------------------------------------------------------------.............................................................

F.1.11 ---------------------------------..T..T.......T.................-.............-................-...-....................................

F.1.12 -------------------------------...............T.........................................................................................

F.1.13 ........................................................................................................................................

F.1.14 ........................................................................................................................................ ........................................................................................................................................

F.1.15 .....--------------------.....................T.........................................................................................

F.1.16 ..............................................T.........................................................................................

F.1.17 ..............................................T.........................................................................................

F.1.18 ................................................................................................................................--------

F.1.19 ..............................................T.........................................................................................

F.1.20 ..............................................T.........................................................................................

F.1.21 ..............................................T......................................................G.................................. .......................................................................................................................................

F.2.1 ...................................G....................................................................................................

F.2.2 ..............................................T......................................................G..................................

F.2.3 ........................................................................................................................................

F.2.4 ........................................................................................................................................

F.2.5 ..............................................T.........................................................................................

F.2.6 ........................................................................................................................................

F.2.7 ........................................................................................................................................

F.2.8 ..............................................T......................................................G..................................

F.2.9 .....T.....................................................................---------------------------..................................

F.2.10 -------------------------.....................T.........................................................................................

F.2.11 -------------------------.....................T.........................................................................................

F.2.12 -------------------------...............................................................................................................

F.2.13 ----------------------------------------......T.A....................T..................................................................

F.3.1 ----------------------------------------......T.A.......................................................................................

F.3.2 -------------------------------------------...T.........................................................................................

F.3.3 ------------------------------------------------A.G.......................-.............................................................

F.3.4 ........................................................................................................................................

F.3.5 ..............................................T.........................................................................................

F.3.6 ..............................................T......................................................G..................................

F.3.7 ..............................................T......................................................G..................................

F.3.8 ........................................................................................................................................

F.3.9 ........................................................................................................................................

F.3.10 ..............................................T.........................................................................................

F.3.11 ........................................................................................................................................

F.3.12 ..........T.............................................................................................................................

F.3.13 ..............................................T.........................................................................................

F.3.14 ------------------------------------------------------------.................................................................-..........

F.3.15 ..............................................T.........................................................................................

**F.4.1** ........................................................................................................................................

**F.4.2** ........................................................................................................................................

**F.4.3** ........................................................................................................................................

**F.4.4** ........................................................................................................................................

**F.4.5** ........................................................................................................................................

**F.4.6** ........................................................................................................................................

**F.4.7** ........................................................................................................................................

**F.4.8** ..................................................................................................A.....................................

**F.4 9** ........................................................................................................................................

**F.4.10** ........................................................................................................................................

**F.4.11** ........................................................................................................................................

**F.4.12** ........................................................................................................................................

**F.4.14** ........................................................................................................................................

**F.4.13** ........................................................................................................................................

**F.4.15** ........................................................................................................................................

**F.4.16** ........................................................................................................................................

**F.4.17** ........................................................................................................................................

**F.4.18** ........................................................................................................................................

**F.4 19** ........................................................................................................................................

**F.4.20** ........................................................................................................................................

**F.5.1** ..................................................................C.....................................................................

**F.5.2** ........................................................................................................................................

**F.5.3** ........................................................................................................................................

**F.5.4** ........................................................................................................................................

**F.5.5** ........................................................................................................................................

**F.5.6** ........................................................................................................................................

**F.5.7** ........................................................................................................................................

**F.5.8** ........................................................................................................................................

**F.5.9** ........................................................................................................................................

**F.5.10** ........................................................................................................................................

**F.5.11** ........................................................................................................................................

**F.5.12** ........................................................................................................................................

**F.5.13** ........................................................................................................................................

**F.5.14** ........................................................................................................................................

**F.5.15** ........................................................................................................................................

Table 1
